# Novel insights in the regulation of CCL18 secretion by monocytes and dendritic cells via cytokines, Toll-like receptors and rheumatoid synovial fluid

**DOI:** 10.1186/1471-2172-7-23

**Published:** 2006-09-19

**Authors:** Antoine WT van Lieshout, Robbert van der Voort, Linda MP le Blanc, Mieke F Roelofs, B Willem Schreurs, Piet LCM van Riel, Gosse J Adema, Timothy RDJ Radstake

**Affiliations:** 1 Department of Rheumatology, Radboud University Nijmegen Medical Centre, The Netherlands; 2 Department of Tumor Immunology, Radboud University Nijmegen Medical Centre, The Netherlands; 3 Department of Hematology, Radboud University Nijmegen Medical Centre, The Netherlands; 4 Department of Orthopedics, Radboud University Nijmegen Medical Centre, The Netherlands

## Abstract

**Background:**

The T cell attracting chemokine CCL18 is produced by antigen presenting cells and a role for CCL18 has been suggested in the pathogenesis of a variety of diseases. Rheumatoid arthritis (RA) is one of these conditions, in which abundant CCL18 production is present. Although Th2 cytokines and IL-10 are known to have an effect on CCL18 production, there are several gaps in our knowledge regarding the exact regulation of CCL18 secretion, both in general and in RA. In this study we provide new insights in the regulation of CCL18 secretion by monocytes and dendritic cells.

**Results:**

In contrast to a large panel of pro-inflammatory stimuli (IL-1β, TNF-α, IL-10, IL-13, IL-15, IL-17, IL-18, IFN-γ), T cell mimicking molecules (RANKL, CD40L) or TLR driven maturation, the anti-inflammatory IL-10 strongly stimulated DC to secrete CCL18. On freshly isolated monocytes, CCL18 secretion was induced by IL-4 and IL-13, in strong synergy with IL-10. This synergistic effect could already be observed after only 24 hours, indicating that not only macrophages and dendritic cells, but also monocytes secrete CCL18 under these stimulatory conditions. A high CCL18 expression was detected in RA synovial tissue and incubation of monocytes with synovial fluid from RA patients clearly enhanced the effects of IL-4, IL-13 and IL-10. Surprisingly, the effect of synovial fluid was not driven by IL-10 of IL-13, suggesting the presence of another CCL18 inducing factor in synovial fluid.

**Conclusion:**

In summary, IL-10 synergistically induces CCL18 secretion in combination with IL-4 of IL-13 on monocytes and monocyte derived cells. The effects of IL-14, IL-13 and IL-10 are strongly enhanced by synovial fluid. This synergy may contribute to the high CCL18 expression in RA.

## Background

Rheumatoid arthritis (RA) is a chronic autoimmune disease that is mainly characterized by inflammation of the synovial tissue (ST), leading to cartilage and bone destruction. Influx of different inflammatory cells into the ST and enhanced production of cytokines and chemokines are all well known features of RA. Chemokines are small proteins that act as key players in the chemo-attraction of different leucocytes and perform their chemo-attractive task through interaction with their receptor on the target cell. Several chemokines have been shown to be abundantly present in RA ST at highly strategic sites [1-3], which suggests a role for these chemokines in the pathogenesis of RA. In this respect, chemokines could be regarded as promising therapeutic targets in RA. This concept has already been translated to the clinic, since the blockade of C Chemokine Receptor 1 (CCR1) has recently been shown to be clinically effective in the treatment of RA [4].

Antigen presenting cells (APC), such as dendritic cells (DC) and macrophages (MΦ), are generally accepted as critical mediators in the complex pathogenesis of RA [5-7]. APC produce a multitude of chemokines that attract specific T cell subsets. Such chemokines are likely to play a critical role in the regulation of immune responses, since they orchestrate the spatial and temporal interaction between APC and T cells, which determines the fate and nature of the immune response. Evidence for this conceptual framework came recently from the observation that blocking APC-T cell interactions using CTLA4-Ig led to a significant reduction of disease activity in RA [8]. Several chemokines orchestrate the attraction of T cells toward DC. It is tempting to speculate that interfering with these chemokines would lead to similar effects on disease activity as the direct blockade of T-cell DC interaction. Of this group of T- cell attracting chemokines, CCL18 and CXCL16 recently came out as potentially interesting targets in RA from previous research by our group and others [9-13].

CC chemokine ligand 18 (CCL18, also DC-CK-1, PARC, AMAC-1) was first identified as a naïve T cell attracting chemokine [14-16]. Next to chemo-attraction, CCL18 plays a role in stimulation of collagen production by fibroblasts [17]. Despite numerous attempts to identify its receptor, CCL18 is still an orphan chemokine. *In vivo*, CCL18 expression was first found in high quantities in the lung, which is caused by the abundant expression by alveolar macrophages [15]. *In vitro*, DC and MΦ have been identified as CCL18 producers [14-16,18,19]. To date, a substantial amount of data points toward the enrichment of DC and MΦ in the synovial tissue which likely to be responsible for the increased levels of CCL18 in RA synovial tissue and synovial fluid (SF) compared with that from healthy individuals [18,20]. In this line, CCL18 has been identified as a clinical marker in Gaucher's disease, a condition in which MΦ activation is likely to play a role in the pathogenesis [21,22]. In addition, a role for CCL18 has been suggested in a large variety of diseases, such as systemic sclerosis and acute lymphoblastic leukaemia [23,24]. In RA, we recently found that circulating CCL18 levels are elevated compared with controls and correlated with disease activity (van Lieshout *et al*. manuscript submitted). Moreover, CCL18 mRNA expression by DC from RA patients was shown to be higher than by DC from healthy controls, which could be influenced by blockade of TNF-α [10,13]. The exact regulation of CCL18 protein secretion however is complicated and the studies published thus far have led to controversial results [18,19,25-27], as elegantly reviewed by Schutyser *et *al [28].

In order to clarify the mechanism of CCL18 expression and secretion in RA, we investigated the role of a large panel of inflammatory mediators known to play a role in the disease process on CCL18 secretion. Here we show that CCL18 secretion by monocytes and DC is regulated by synergistic effects between IL-4/IL-13, IL-10 and RA SF, whereas pro-inflammatory cytokines and Toll-like receptor (TLR) ligands did not have any influence on CCL18 secretion. These data add novel information to the puzzle of increased CCL18 expression in RA.

## Results

### IL-10 strongly enhances CCL18 production by moDC while maturation and pro-inflammatory mediators do not

**Figure 1:**
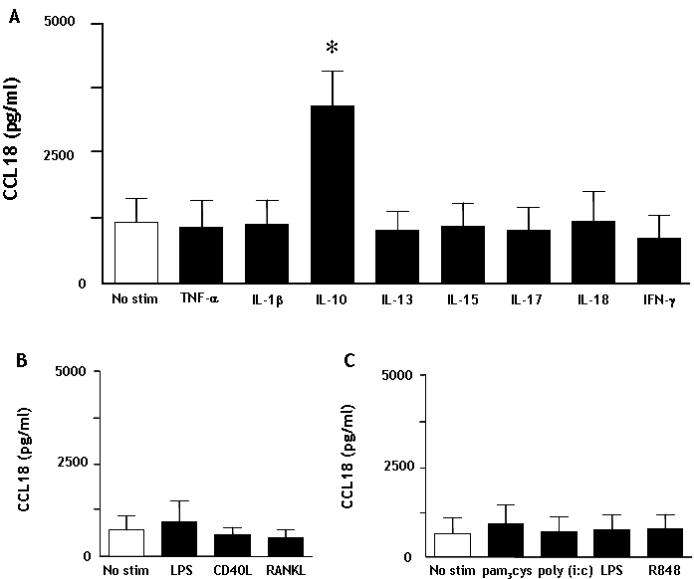
**IL-10 induces CCL18 secretion by monocyte derived dendritic cells**. Panel A depicts CCL18 secretion by MoDC (cultured with IL-4 and GM-CSF) upon stimulation with TNF-α, IL-1β, IL-10, IL-13, IL-15, IL-17, IL-18 (all 20 ng/ml) and IFN-γ (10 ng/ml) (n = 6). Panel B depicts CCL18 secretion by MoDC upon stimulation with LPS (2 μg/ml), CD40L or RANKL (20 ng/ml) (n = 3). Panel C depicts CCL18 secretion upon stimulation upon TLR2 (pam_3_cys, 10 μg/ml), TLR3 (poly (i:c), 25 μg/ml), TLR4 (LPS, 2 μg/ml) or TLR7/8 (R848, 1 μg/ml) mediated stimulation (n = 5) In all experiments, a direct comparison was made with unstimulated cells. The bars represent the mean (± SEM) CCL18 secretion in pg/ml. * represents a p-value of <0,05 (Wilcoxon Signed Rank test)

**Figure 2:**
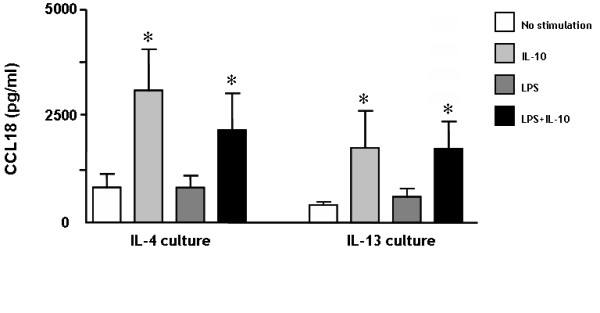
**Similar pattern of CCL18 production by IL-4 vs. IL-13 cultured monocyte derived dendritic cells**. Immature MoDC were initially cultured with either IL-4 or IL-13, in combination with GM-CSF. On day 6, these immature DC were stimulated for 48 hours with IL-10 (20 ng/ml), LPS (2 μg/ml) or both. The bars represent the mean (± SEM) CCL18 (pg/ml) production/ml of 6 individual experiments. * represents a p-value of <0,05 (Wilcoxon Signed Rank test)

First we investigated whether several mediators that are known to be important in RA were able to enhance CCL18 production by MoDC. In line with previous studies, unstimulated immature DC (iDC) produced significant amounts of CCL18 [19]. Interestingly, incubation with TNF-α, IL-1β, IL-13, IL-15, IL-17, IL-18 and IFN-γ did not stimulate CCL18 secretion when added to day 6 iDC (n = 6). In contrast, the anti-inflammatory IL-10 strongly induced CCL18 production by iDC (p = 0.03) (figure 1a). Next we tested whether factors well known to induce maturation or T cell mimicking could induce CCL18 production. These experiments demonstrated that LPS, CD40L and RANKL did not enhance CCL18 production (n = 3) (figure 1b). Recent studies demonstrated that other TLR pathways than TLR4 are all capable of inducing DC maturation, but have different effects on cytokine production [29-31]. However, stimulation of TLR2 (pam_3_cys), TLR3 (poly (i:c)), TLR4 (LPS) or TLR7/8 (R848) did not sort any effect on CCL18 secretion (n = 6) (figure 1c), whereas they did elicit a potent cytokine response [31]. Since IL-13 is more abundantly present in RA than IL-4 and since some conflicting results have been published on CCL18 production induced by LPS when DC were cultured with IL-13 vs. IL-4, we compared these culture methods (n = 6). In both the IL-4 and IL-13 cultures, IL-10 strongly induced CCL18 (p = 0.03 for both IL-4 and IL-13 culture), while LPS again did not (figure 2). In addition, IL-10 in combination with LPS was not significantly different from IL-10 alone (figure 2). Also co-stimulation with LPS and the cytokines tested (as in figure 1) did not sort any effect on CCL18 secretion (data not shown).

### IL-10 acts in synergy with IL-4/L-13 in promoting CCL18 production by monocytes

**Figure 3:**
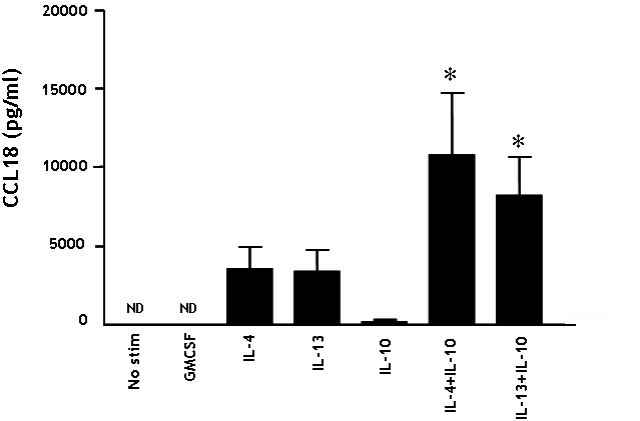
**Synergistic effect on CCL18 secretion by monocytes upon stimulation with IL-4/IL-13 in combination with IL-10**. MACS isolated monocytes were cultured for 6 days and stimulated on day 1 with IL-4 (500 U/ml), IL-13 (20 ng/ml), IL-10 (20 ng/ml) or a combination of the cytokines. The bars represent the mean (± SEM) CCL18 (pg/ml) of 6 individual experiments. In all experiments, a direct comparison was made with with unstimulated cells. ND = not detectable. * represents a p-value of <0,05 (Wilcoxon Signed Rank test)

**Figure 4:**
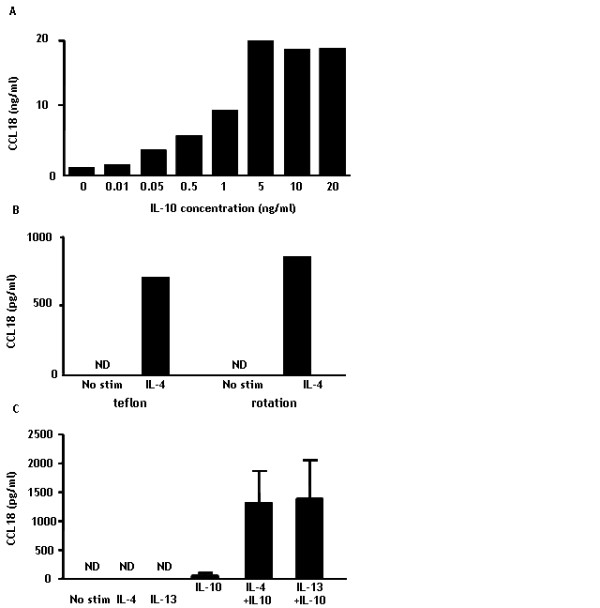
**Synergistic CCL18 production by monocytes upon stimulation with IL-4 and IL-10 can be induced rapidly and by low concentrations of IL-10**. Panel A depicts CCL18 secretion by monocytes upon stimulation with different doses of IL-10 in the presence of IL-4. Panel B represents CCL18 secretion by monocytes that were cultured for three days in the presence or absence of IL-4 in teflon bags or rotation discs to prevent adherence. Panel C depicts CCL18 secretion by monocytes that were cultured for 24 hours with no stimulation or in the presence of IL-4, IL-13, IL-10 or a combination of IL-4/IL-13 with IL-10. The bars in panel A and B represent the mean CCL18 (pg/ml) of duplicates of 1 individual experiment and panel C shows the mean (± SEM) of 3 separate experiments. ND = not detectable

MoDC and alternatively activated MΦ (AaMΦ) [32,33] are known to produce CCL18. Both these cell types originate from CD14+ monocytes and depend on IL-4 or IL-13 (in combination with GM-CSF for MoDC) for their differentiation. To determine whether CCL18 secretion by myeloid cells is dependent on these cytokines, monocytes were freshly isolated and stimulated with GM-CSF, IL-4, IL-13 and IL-10 alone or in combinations (n = 6). Even after 6 days, unstimulated and GM-CSF treated monocytes/macrophages did not secrete CCL18, whereas both IL-4 and IL-13 stimulation resulted in a clear secretion of CCL18, which is in line with previous findings on AaMΦ [16]. Interestingly, stimulation with IL-10 alone only had a minimal effect on CCL18 production by these monocytes/macrophages. When IL-10 was provided together with IL-4 or IL-13, this resulted in 3- and 2-fold increase in CCL18 secretion respectively (figure 3). Interestingly, already in low concentrations, IL-10 had its synergistic effect with IL-4 (figure 4a). In order to rule out effects of adherence, we cultured CD14+ monocytes/macrophages for three days in teflon bags [34] and in rotation discs [35]. The morphology of these cells was comparable with freshly isolated monocytes according to their forward/side scatter pattern (data not shown). In both cultures, IL-4 did still induce CCL18 production in the same way as the cultures in 24-wells plates (figure 4b). As a proof of principle, we next tested whether the synergy between IL-4/IL-13 and IL-10 could already be observed after only 24 hours. Intriguingly, we could indeed observe a clear CCL18 secretion after 24 hours upon stimulation of freshly isolated monocytes with IL-4/IL-13 and IL-10, whereas stimulation with IL-4, IL-13 or IL-10 alone did only result in a minor or even undetectable CCL18 secretion (n = 3) (figure 4c). Since IL-10 appeared to synergize with IL-4 and IL-13, we investigated whether these cytokines could up-regulate each other's receptors, possibly resulting in enhanced signaling. This was not the case; IL-10 did not up regulate either the IL-4/IL-13 common receptor IL-4Rα1 or the specific IL-13Rα2. Furthermore, IL-4 had no effects on IL-10Rα (data not shown).

### RA synovial fluid enhances CCL18 secretion independently of IL-10 and IL-13

**Figure 5:**
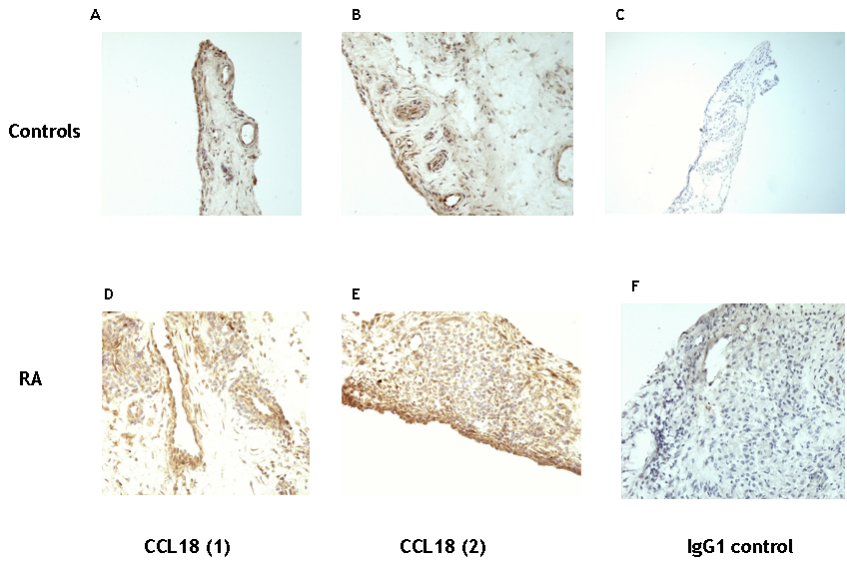
**CCL18 expression in normal and RA synovial tissue**. Panel A and B depict 2 sections of control synovium, where CCL18 expression is expressed in parts of the lining and some perivascular regions. Panel D and E depict 2 representative synovial sections from RA where CCL18 is present in the lining and perivascular regions. Panel C and F represent isotype controls on RA synovium and that from healthy individuals respectively.

**Figure 6:**
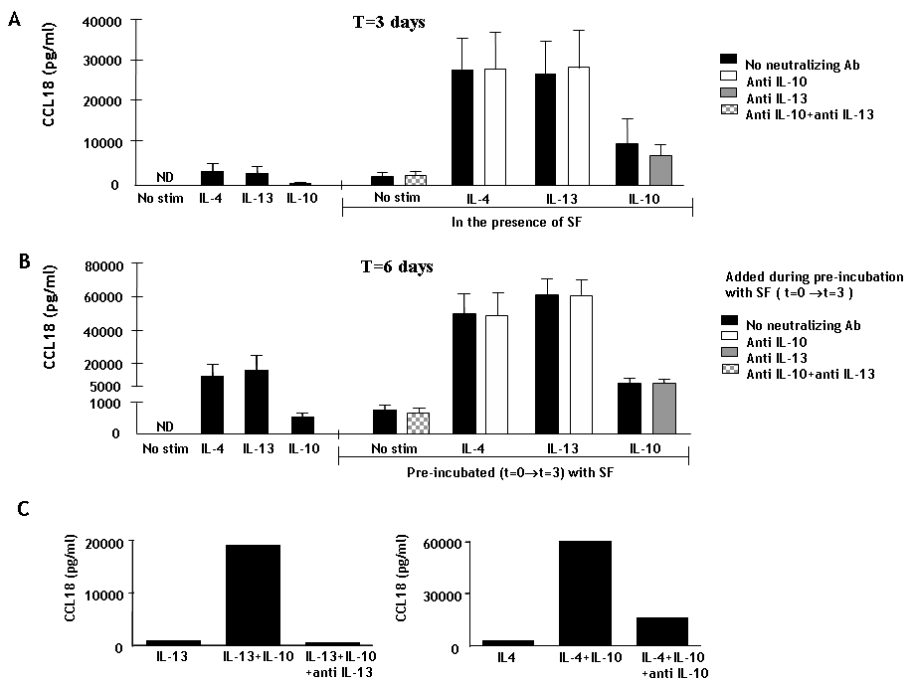
**CCL18 production by monocytes upon stimulation with RA synovial fluid**. Panel A. Monocytes/macrophages were cultured for three days. A part of the cells were incubated with IL-10, IL-4 or IL-13 alone (left side of the figure) and another part was incubated with these cytokines in the presence of RA SF (right side of the figure). Half of the latter were also incubated with neutralizing antibodies against IL-10 and IL-13 or both, which is shown by the white, gray and checked bars respectively in panel A. The bars represent the mean CCL18 pg/ml from 6 separate experiments. ND = not detectable. Panel B. The cells were then washed and only the cytokines were added again to the fresh medium. After another 3 days, supernatant was measured again. The left side of the figure shows the CCL18 production upon stimulation with IL-4, IL-13 and IL-10. The right side shows the production upon stimulation with these cytokines by cells that have been pre-incubated with SF for three days, in the presence or absence of anti IL-10, anti IL-13 or both (white, gray and checked bars respectively). ND = not detectable. * represents a p-value of <0,05 (Wilcoxon Signed Rank test). Panel C. The potency of neutralizing antibodies against IL-13 and IL-10 was tested by investigating their ability to inhibit the synergy between IL-13 and IL-10 and IL-4 and IL-10. The bars represent the mean (± SEM) CCL18 secretion (pg/ml).

We and others demonstrated CCL18 expression in RA ST in the lining and the peri-vascular regions [10,20]. In figure 5, we show a high CCL18 expression in RA synovial tissue (figure 5a,b,c), which was preferentially located in both the synovial lining layer and the peri-vascular regions. Intriguingly, CCL18 was also expressed in control synovial tissue, although not as abundant as in RA ST (figure 5d,e,f). Notably, some parts of the sections were even negative for CCL18, which is in sharp contrast with RA. In order to explain the abundant CCL18 expression in RA, we tested whether incubation with RA SF could induce CCL18 production on monocytes/macrophages. Since RA SF itself contains CCL18 [18,20], we cultured freshly isolated monocytes for 3 days in the presence of SF, washed the cells and cultured on for another 3 days in the absence of RA SF (n = 6). Firstly, this pre-incubation with RA SF resulted in marked CCL18 production (mean 676 (± 151) pg/ml) (figure 6b). Secondly, culture of freshly isolated monocytes in the presence of RA SF, resulted in a 9- and 10-fold increase in CCL18 secretion upon stimulation with IL4/IL-13 respectively and a 22-fold increase compared with IL-10 alone (figure 6a). Intriguingly, this synergistic effect with IL-4, IL-13 and IL-10 could still be observed after 3 days of culture in the complete absence of RA SF (figure 6b), indicating that the cell does not require a continuous stimulation in order to secrete CCL18. To exclude intrinsic differences between monocytes/macrophages from RA patients and controls may contribute to the effects on CCL18 secretion, we tested whether monocytes/macrophages from RA patients (n = 3) responded differently to combinations of IL-4, IL-13, IL-10 and SF. No difference in the CCL18 secretion pattern was observed between monocytes/macrophages of healthy controls and RA patients upon these stimuli (data not shown), ruling out intrinsic differences in monocytes in RA that affect CCL18 secretion.

Since the synergy caused by RA SF appeared similar to the synergy between cytokines we already observed (figure 4), we tested whether IL-10 and/or IL-13, both present in RA SF, were responsible for this phenomenon by blocking these cytokines with neutralizing antibodies. The potency of these antibodies was first tested by determining their ability to inhibit the synergistic CCL18 secretion upon stimulation with a combination of IL-10 and IL-4/IL-13. Addition of anti-IL-10 resulted in a 73% inhibition of the synergy between IL-4 and IL-10 and anti IL-13 completely abrogated the synergistic effect of IL-13 with IL-10 (figure 6c). Unexpectedly however, blockade of IL-10 or IL-13 in SF in the presence of IL-4 or IL-13 and IL-10 respectively did not inhibit the synergy between these cytokines and SF (figure 6a&b), suggesting that SF contains a yet unidentified factor that triggers CCL18 secretion.

## Discussion

In this study, we add new pieces to the complicated puzzle of CCL18 regulation in RA. Firstly, we demonstrate that CCL18 production can be induced by IL-4, IL-13 and IL-10 in monocyte derived cells. Secondly, we show that a large panel of pro-inflammatory stimuli and TLR mediated signals leading to DC maturation are of no influence on CCL18 production. Thirdly, IL-10 only induces a minor CCL18 secretion, but acts in synergy with both IL-4 and IL-13 on monocytes and monocyte derived cells. Finally, we provide evidence that RA SF is able to induce CCL18 secretion in strong synergy with IL-4, IL-13 and IL-10, which could not be inhibited by a blockade of IL-10 and IL-13.

CCL18 can be produced by MoDC as well as by certain types of MΦ. Often these cell types are considered to be totally different cells. However, the differences between MoDC and AaMΦ are not that large, since monocyte derived macrophages are cultured in the presence of GM-CSF by some groups [36] and both cells require the presence of IL-4 or IL-13. Penna and co workers demonstrated that several *in vivo *DC subtypes were not able to produce CCL18 [37], which is in contrast with previous findings, where CCL18 mRNA expression was found on CD11c+ myeloid blood DC [27]. Moreover, *in vitro *cultured MoDC have been identified as potent CCL18 producers [18,19]. These data suggest that a CD14+ monocyte origin in combination with a stimulation by IL-4/IL-13 is critical for CCL18 secretion. This hypothesis is strengthened our data, demonstrating that non-adherent monocytes/macrophages were able to produce CCL18 under the influence of IL-4. In addition, the synergistic effects of IL-4/IL-13 and IL-10 on CCL18 secretion by freshly isolated monocytes were already clearly visible after 24 hours. This indicates that a full differentiation into DC or MΦ is not essential for CCL18 production as has been suggested previously for CCL18 mRNA expression [16]. Thus monocytes rapidly secrete CCL18 upon triggering with the right stimuli.

In the literature there is still some controversy regarding the effect of DC maturation on CCL18 production. Vulcano and co workers suggested that DC down regulate their CCL18 secretion upon maturation [19]. This is in contrast with results from other studies, where maturation caused an increased mRNA expression [10,26,27]. A similar contrast between protein and mRNA was found on blood DC [27,37]. The reason for these differences between mRNA expression and protein secretion patterns still needs to be investigated in detail. Recently, we already provided evidence that DC maturation does not influence CCL18 protein secretion [18], which is further strengthened by the data from the present study, in which different TLR stimulatory pathways did not induce CCL18 production, whereas full DC maturation was achieved [31]. Also TNF-α and CD40L, both well appreciated inducers of DC maturation [38,39], did not enhance CCL18 production. Perhaps the discrepancy between the different reports is hidden in subtle differences in culture conditions, which are difficult to trace in the published data. Intriguingly, stimulation with IL-10 alone only lead to a marginal induction of CCL18 secretion by monocytes/macrophages, but did act in a strong synergy with IL-4 or IL-13. The latter is not caused by an up regulation of the receptors IL-10Rα, IL-4Rα or IL-13Rα2 (data not shown). Probably intracellular pathways direct the synergy between these cytokines, which is an interesting topic that warrants further investigation.

We showed that RA SF induces CCL18 production and strongly synergizes with IL-4, IL-13 and IL-10. Blocking studies revealed that neither IL-10 nor IL-13 in SF were responsible for this effect. This suggests the presence of another, yet unidentified CCL18 inducing factor in RA SF. Another explanation for this fact might be the presence of inhibiting factors in SF that counter-regulate the effects of IL-10 and IL-13. The identification of the factor in SF that drives the effects on CCL18 secretion may provide important new insights to the pro-vs. anti inflammatory balance in RA. In order to find this factor in a complex fluid like SF, more knowledge on the pathways of CCL18 regulation is critical. Another intriguing observation from our study is the finding that pre-incubation with SF lead to a sustained synergistic CCL18 secretion upon stimulation with IL-4, IL-13 and IL-10. This could be regarded as an "imprinting effect", meaning that the cell's previous environment determines the nature of response to stimuli, even when the cell is no longer in such an environment. Results from previous studies, in which we showed that moDC from RA patients differ in phenotype and cytokine response from control DC after 6 days in culture might also be explained by such a phenomenon [40,41].

Upon the encounter of an antigen, DC normally mature and migrate to lymphoid tissues in order to perform their task of antigen presentation to T cells. Immature DC or MΦ can also encounter naïve T cells in the periphery, which subsequently might result in tolerance [42]. This peripheral tolerance is a critical mechanism to prevent auto-immunity. A role for CCL18 in this part might explain the high expression of CCL18 by alveolar MΦ [15,16], which are located at a site where the maintenance of tolerance to non-pathogenic antigens, that are constantly present, is crucial. Also the synergistic effect on CCL18 secretion that we found with IL-10, a cytokine that is well appreciated as a pivotal regulator of the immune system, fits in this picture. The synovial lining in the joints has similarities with the alveolar lining in the lung. They both consist of MΦ-like cells and both form a barrier to a site in which self- and non-pathogenic antigens are constantly present. The disease process in RA is considered to be driven by pro inflammatory cytokines such as IL-1β, TNF-α, IL-17 and IL-18 [43-48], whereas CCL18 is regulated by IL-10, IL-4 and IL-13. It is therefore tempting to speculate that the high CCL18 expression in RA is designed to uphold peripheral tolerance, which however seems to fail. This failure might be explained in two ways. The first explanation might be that the skewing in the balance towards Th1 is still present despite the upregulation of anti inflammatory mediators. Secondly, mature DC are present in the synovial tissue in perivascular regions and secondary lymphoid organs [3,49], which is in sharp contrast with healthy synovial tissue. Therefore an explanation for the ongoing immune process might be that these mature DC direct naïve T cells towards a phenotype that drives the pro-inflammatory immune response in the synovial tissue.

## Conclusion

In summary, we provide evidence that monocyte derived cells produce CCL18 under the influence of IL-4 and IL-13. IL-10 acts in strong synergy with IL-4 and IL-13 as a key regulator of CCL18 production by monocytes, which indicates that CCL18 secretion is not confined to fully developed DC and MΦ. In addition, the effects of IL-4, IL-13 and IL-10 are strongly enhanced by RASF, which is due to yet unidentified factors. Both the *in vivo *expression pattern and the contributing factors to its regulation *in vitro *are suggestive for a role for CCL18 in the regulation of the immune system, both in health and auto-immune diseases such as RA.

## Methods

### Patients and samples

For cell culture experiments, 50 ml peripheral blood was taken from healthy volunteers and RA patients after receiving informed consent in 10 ml lithium heparine (Vacutainer, USA) tubes. Synovial biopsies from RA patients were taken with small needle arthroscopy (Storz, Tutlingen, Germany). Synovial fluid from RA patients was obtained during arthroscopy. For our experiments in which monocytes were stimulated with SF, a pool of SF from 10 different RA patients was used. Synovial samples from healthy controls were taken during scheduled arthroscopic procedures by orthopedic surgeons in patients with traumatic knee injuries. The Nijmegen medical ethics committee (MEC) approved these studies.

### Recombinant proteins and antibodies

For stimulation of iDC, we used 20 ng/ml recombinant (rh) IL-1β, rhTNF-α, rhIL-10, rhIL-13, rhIL-15, rhIL-17, rhIL18, 10 ng/ml IFN-γ (all R&D systems, Minneapolis, USA), or 20 ng/ml RANKL and CD40L (Pepro Tech, Rocky Hill, USA). DCs were cultured with 500 U/ml IL-4 and 800 U/ml GM-CSF. The same IL-4 concentration was used for monocyte stimulations. For Toll-like receptor stimulation, 10 μg/ml pam_3_cys (TLR2), 25 μg/ml poly (i:c) (TLR3), 2 μg/ml lipopolysacharide (LPS) (TLR4), or 1 μg/ml R848 (TLR7/8) was used [31]. Blockade of IL-10 (Ebioscience, San Diego, USA) and blockade of IL-13 (Diaclone, Becanson, France) was achieved with a 1000× excess of a neutralizing antibody. For FACS analysis, we used mouse-anti human antibodies against CD14, (Dako, Glostrup, Denmark), CD83 (Beckman Coulter, Mijdrecht, The Netherlands), IL-4Rα (Santa Cruz, California USA), IL-13Rα II (R&D systems, Minneapolis, USA) and IL-10Rα (R&D systems, Minneapolis, USA) or mouse-isotype control (goat IgG for IL-13RαII). For ELISA, mouse anti-human and biotynilated goat anti-human CCL18 were used as capture and detection antibody (R&D systems, Minneapolis, USA). A standard curve was made with rhCCL18 (R&D systems, Minneapolis, USA). Immuno histochemistry for CCL18 was performed with AZN-CK18B [18] as a primary antibody.

### Monocyte/macrophage and MoDC isolation and culture

MoDC were cultured using essentially the same protocol as described previously [13,40]. In brief, peripheral blood mononuclear cells were isolated from venous blood by density gradient centrifugation over Ficoll-Hypaque (Amersham Biosciences, Roosendaal, The Netherlands). The interphase was collected and washed with citrated phosphate buffered saline, and the cells were allowed to adhere for 1 hour at 37°C in RPMI-1640 (Life Technologies, Breda, The Netherlands) supplemented with 2% human serum in culture plates (Costar, Badhoeverdorp, The Netherlands). Adherent cells were cultured in RPMI-1640 Dutch modification supplemented with 10% fetal calf serum L-glutamine (Life Technologies, Breda, The Netherlands) and antibiotic-antimycotic agents (Life Technologies, Breda, The Netherlands) (culture medium) in the presence of IL-4 (500 U/ml; Strathmann Biotech, Hamburg Germany) and granulocyte monocyte-colony stimulating factor (GM-CSF) (800 U/ml; R&D systems, Minneapolis USA) for 6 days. Fresh culture medium with the same supplements was added at day 3, and then iDC were harvested at day 6. To generate mature DC, immature DC were re-suspended in a concentration of 0,5 × 10^6^/ml in fresh IL-4 and GM-CSF containing culture medium. Immature DC were then stimulated with cytokines or maturation stimuli in the presence of IL-4 and GM-CSF. DC were harvested after another 48 hours of culture. For CCL18 measurements in supernatant of cells stimulated with TLR ligands, aliquoted culture supernatant from previous experiments was used [31].

For the culture of monocytes/macrophages, CD14+ cells were isolated with magnetic cell sorting and separation (MACS). In brief, mononuclear cells were labelled with anti CD14 microbeads (Miltenyi Biotec, Amsterdam, the Netherlands) and incubated for 30 minutes at 4°C. CD14 positive cells were then separated from the other cells using a MACS column (Miltenyi Biotec, Amsterdam, the Netherlands) according to the manufacturers instructions. CD14+ cells were cultured in a concentration of 0,5 × 10^6 ^cells/ml in culture medium for up to 6 days. Where appropriate, fresh culture medium was added on day 3. After 6 days, supernatant was collected for ELISA and cells were prepared for FACS analysis. In some additional experiments, monocytes/macrophages were cultured for three days in the same concentration and in the same media in teflon bags [34] or in rotation discs (Cellon, Luxembourg) [35] to prevent adherence of the cells. In experiments in which monocytes/macrophages were stimulated with RA SF, the cells were cultured for three days in the presence of 100 μl RA SF. Cells were then harvested and re-suspended in fresh culture medium without SF, but with the cytokines that were present in the first three days. Anti-IL-10 or anti-IL-13 neutralizing antibodies were only present during the first three days.

### Immuno histochemistry

For immuno histochemistry, frozen ST was cut into 7 μm sections and mounted on slides, air-dried, and stored at -80°C. Before staining, the cryosections were air-dried, fixed in acetone for 10 min and air-dried again. The sections were then stained with 5 μg/ml mouse anti human CCL18 or isotype control at 37°C for 1 hour at room temperature (RT) and washed in PBS. Endogenous peroxidase was blocked with 0,3% H_2_O_2_/methanol. After another wash-step, the sections were incubated with a biotin-conjugated horse anti-mouse antibody at RT for 30 min. Next, the samples were washed and incubated with avidin-biotin-HRP complex (Vector, Burlingham, UK) at RT for 20 min. Next, the section were stained with diaminodenzidine (DAB) (Sigma, Zwijndrecht, the Netherlands). Finally, sections were then counterstained with hematoxylin, rehydrated and mounted in to allow microscopic evaluation of the samples.

### Fluorescence-Activated Cell Sorter (FACS) analysis

The phenotype of cells was characterized by using flow cytometry techniques (FACS). For this aim, cells were harvested and collected by means of centrifugation and further processed on melting ice. Cells were diluted in buffer solution (PBS with 1% bovine albumine, pH 7,4) in a concentration of 1.10^6 ^cells/ml and plated in v-shaped 96 wells plates (1.10^5 ^cells per plate). Cells were labeled with monoclonal mouse- or goat anti human antibodies or mouse-isotype control (goat IgG for IL-13RαII) and incubated at a temperature of 4°C for 45 minutes. Cells were then washed and labeled with goat-anti-mouse (or rabbit anti-goat when appropriate) FITC (Zymed Laboratories, South San Francisco, USA) as a secondary antibody. After another 30 minute incubation at 4°C, cells were again washed, diluted in buffer solution and transferred into FACS tubes. Cells were gated according to their forward and side scatters and fluorescence was measured with a FACSCalibur^® ^(Becton-Dickinson, San Jose, USA) and Cellquest^® ^software.

### Enzyme Linked Immuno Sorbent Assay (ELISA)

For the detection of CCL18 protein levels of CCL18 in supernatant, a sandwich ELISA was performed as described previously [18,50]. In brief, maxisorb ELISA plates (Nunc, Roskilde, Denmark) were coated overnight with 50 μl/well 1 μg/ml capture antibody in PBS. Next, the plates were washed 3 times with PBS and blocked with 300 μl 1% Bovine Albumin (Sigma, Zwijndrecht, the Netherlands) in PBS for a minimum of 1 h at RT. After washing 3 times with ELISA wash buffer (PBS containing 0.05% Tween-20), the plates were incubated with 50 μl/well of serial dilutions of the sample for 2 hrs at RT. Serial dilutions of rhCCL18 were used to obtain a standard curve. After washing 3 times with ELISA wash buffer, the plates were incubated with 50 μl/well of 0.05 μg/ml secondary antibody at RT for 1 hr. Thereafter, the plates were washed 3 times with ELISA wash buffer, and incubated with 50 μl/well of streptavidin conjugated to Poly-Horse Radish Peroxidase (CLB, Amsterdam) for 20 minutes at RT. After washing 3 times with ELISA wash buffer, the presence of HRP was detected using 50 μl/well 3,3',5,5-tetramethylbenzidine (TMB) (Biomerieux, Marcy l'Etoile, France) diluted in peroxide buffer (UP) (Biomerieux, Marcy l'Etoile, France). The reaction was stopped with 50 μl/well 2,5M H_2_SO_4_. Absorbance was measured at 450 nm using a Magellan Tracker V4.XX (Tecan Austria GMBH). As an internal control for inter-assay variability, a sample of pooled normal human serum (n = 300) was taken along in all assays. The maximal accepted inter-assay variability is 10%. The detection limit of the ELISA is 100 pg/ml.

### Statistical analysis

CCL18 production levels by monocyte derived cells upon different stimulations were compared with a Wilcoxon Signed Rank test. P-values < 0,05 were considered significant.

## Abbreviations

C Chemokine Ligand 18 (CCL18), Chemokine (CK), Rheumatoid Arthritis (RA), Antigen presenting cells (APC), Dendritic cell (DC), Macrophage (MΦ), immature DC (iDC), mature DC (mDC), Synovial Tissue (ST), Synovial Fluid (SF), Toll-like receptor (TLR), Chemokine Receptor 1 (CCR1), Alternatively activated MΦ (AaMΦ), Monocyte derived dendritic cell (MoDC), Fluorescence-Activated Cell Sorter (FACS), Enzyme Linked Immuno Sorbent Assay (ELISA).

## Competing interests

The author(s) declare that they have no competing interests.

## Authors' contributions

AvL performed the experiments, provided the RA synovial biopsies, designed the study and wrote the manuscript. RvdV co-designed the study and edited the manuscript. LlB performed and co-designed experiments. MR performed and designed all TLR experiments. BS provided synovial tissue from controls and edited the manuscript. PvR supervised the study and edited the manuscript. GA co-designed the study and edited the manuscript. TR co-designed the study and edited the manuscript. All authors read and approved the manuscript.
